# Gut microbiome in colorectal cancer: metagenomics from bench to bedside

**DOI:** 10.1093/jncics/pkaf026

**Published:** 2025-03-05

**Authors:** Amir Torshizi Esfahani, Nikta Zafarjafarzadeh, Fatemeh Vakili, Anahita Bizhanpour, Amirhesam Mashaollahi, Bita Karimi Kordestani, Mahdieh Baratinamin, Somayeh Mohammadpour

**Affiliations:** Basic and Molecular Epidemiology of Gastrointestinal Disorders Research Center, Research Institute for Gastroenterology and Liver Diseases, Shahid Beheshti University of Medical Sciences, Tehran, Iran; Department of Cellular and Molecular Biology, Faculty of Advanced Science and Technology, Medical Sciences, Islamic Azad University Tehran, Tehran, Iran; Department of Cellular and Molecular Biology, Faculty of Advanced Science and Technology, Medical Sciences, Islamic Azad University Tehran, Tehran, Iran; Department of Cellular and Molecular Biology, Faculty of Advanced Science and Technology, Medical Sciences, Islamic Azad University Tehran, Tehran, Iran; Department of Cellular and Molecular Biology, Faculty of Advanced Science and Technology, Medical Sciences, Islamic Azad University Tehran, Tehran, Iran; Department of Cellular and Molecular Biology, Faculty of Advanced Science and Technology, Medical Sciences, Islamic Azad University Tehran, Tehran, Iran; Department of Cellular and Molecular Biology, Faculty of Advanced Science and Technology, Medical Sciences, Islamic Azad University Tehran, Tehran, Iran; Basic and Molecular Epidemiology of Gastrointestinal Disorders Research Center, Research Institute for Gastroenterology and Liver Diseases, Shahid Beheshti University of Medical Sciences, Tehran, Iran

## Abstract

Colorectal cancer (CRC) is a major global health challenge. Emerging research highlights the pivotal role of the gut microbiota in influencing CRC risk, progression, and treatment response. Metagenomic approaches, especially high-throughput shotgun sequencing, have provided unprecedented insights into the intricate connections between the gut microbiome and CRC. By enabling comprehensive taxonomic and functional profiling, metagenomics has revealed microbial signatures, activities, and biomarkers associated with colorectal tumorigenesis. Furthermore, metagenomics has shown a potential to guide patient stratification, predict treatment outcomes, and inform microbiome-targeted interventions. Despite remaining challenges in multi-omics data integration, taxonomic gaps, and validation across diverse cohorts, metagenomics has propelled our comprehension of the intricate gut microbiome-CRC interplay. This review underscores the clinical relevance of microbial signatures as potential diagnostic and prognostic tools in CRC. Furthermore, it discusses personalized treatment strategies guided by this omics’ approach.

## Background

Colorectal cancer (CRC) poses a substantial global health challenge, ranking as the third most common cancer worldwide^1^ and the second leading cause of cancer-related mortality.^2^ A promising decline in CRC mortality is noted, however, concerns persist. These include a troubling demographic shift toward younger individuals (aged younger than 50 years), an increased incidence of advanced-stage diagnoses, and a perplexing shift from right-sided to left-sided tumors despite the advantages of screening. These changes may be influenced by as-yet-unknown factors.^3^

CRC is a multifaceted disease characterized by genetic and environmental factors, advancing through stages like polyp formation, adenoma, and ultimately resulting in a malignant tumor.^4^ Despite extensive research, challenges remain, including the lack of novel biomarkers and tumor diversity. The Human Genome Project has led to a shift in understanding CRC through omics sciences like transcriptomics, proteomics, metagenomics, metabolomics, and radiomics. This approach advances understanding of CRC progression and allows for molecular subtype categorization, potentially improving patient stratification.^5^

Numerous studies have provided evidence supporting the connection between CRC, the gut microbiota, and the metabolites they generate.^6^^,^^7^ Gut bacteria produce metabolites affecting host metabolism and immune responses, while disruptions may increase the production of cancer-causing substances.^8^ The gut microbiota can also induce inflammation, influence cell cycle kinetics, and prompt DNA damage in colonic epithelial cells, potentially contributing to cancer initiation and progression.^9^

The human gut contains a gastrointestinal microbiota, consisting of roughly 40 trillion microorganisms.^10^ Advancements in high-throughput environmental sequencing techniques have enabled a comprehensive understanding of the microbial ecosystem within the gastrointestinal tract.^11^ Metagenomics constitutes a DNA sequencing methodology employed to investigate the intricate microbial community within the gastrointestinal tract.^12^ Fecal metagenomics is a promising noninvasive diagnostic tool for quantifying gut microbiome,^13^ identifying early onset CRC patients,^14^ and demonstrating a strong correlation between gut microorganisms and cancer treatment effectiveness.^15^ During the progression of CRC, gut microbiota structure undergoes dynamic shifts in microbial composition, gene abundance, and metabolite profiles, potentially impacting the oncogenic microenvironment.^7^

In this review, we extensively examine the developing field of CRC research, particularly emphasizing the transformative possibilities brought by metagenomics. Our objective is to elucidate the pivotal role these cutting-edge methodologies can play in enhancing CRC screening, early detection, progression tracking, and response to anticancer therapies.

## Colorectal cancer and the gut microbiota

### Overview of the human gut microbiome

The vibrant ecosystem known as the human gut microbiota, sometimes labeled the “forgotten organ,” thrives within the extensive gastrointestinal tract, with a particularly high presence in the colon.^10^^,^^16^^,^^17^ Consisting of trillions of microorganisms such as bacteria, viruses, fungi, and other microbes, the gut microbiome coordinates an intricate web of interactions that reach far beyond the boundaries of the gut.^18-20^ The gut microbiota is a crucial participant in various physiological processes, including digestion, nutrient absorption, and immune system modulation.^21-23^ Maintaining a state of equilibrium is essential for gut health and the well-being of the host.^24-26^

*Gut dysbiosis* is a term used to describe compositional and functional changes that occur because of an imbalance between symbiotic and opportunistic microbes in the gut.^27^ This dysregulation can occur because of the reduction of beneficial microbes, the overgrowth of harmful microbes, and a decrease in overall microbial diversity.^28^ Environmental factors primarily shape the composition and structure of the gut microbiota, with only a minimal 1.9% of the gut microbiome being attributed to hereditary factors.^29^ Individual differences exist in the composition of gut microbiota, which remains relatively stable within a single individual.^30^ Host-specific factors, such as genetic predisposition, health status, and lifestyle choices, along with external environmental factors like diet, antibiotics, drugs, and hygiene practices, contribute significantly to the emergence of dysbiosis.^31^

### Gut microbiota in CRC

The human colon, one of the most densely inhabited microbial ecosystems,^10^^,^^32^ hosts approximately 30 trillion bacteria, engaging in continuous communication with the intestinal epithelium, immunological cells, and the mucosal barrier.^11^^,^^33^ Dysbiosis can trigger inflammation, alter the production of harmful substances, and influence the cancer-promoting environment in the colon and rectum.^34^^,^^35^ Research indicates distinct gut microbiota in individuals with CRC, characterized by lower abundance of protective taxa (eg, *Roseburia intestinalis*) and higher abundance of procarcinogenic bacteria (eg, *Bacteroides*, *Escherichia*, *Fusobacterium*, and *Porphyromonas*).^30^^,^^36^ Disparities are also observed in the fecal and mucosal microbiota,^37^ and dysbiosis has been linked to CRC development and progression.^38-43^

The gut microbiota plays a crucial role in the development and progression of CRC through various molecular processes, including promoting mutagenesis through genotoxins, adjusting oncogenic signaling pathways, initiating inflammation, facilitating immune avoidance, integrating host and dietary elements, and interacting with host genetics.^19^^,^^30^^,^^44^ Moreover, it plays a role in shaping the response of CRC patients to chemopreventive and therapeutic drugs by modulating drug bioavailability, developing chemoresistance, enhancing antitumor immunity, and adjusting chemotherapy effectiveness while minimizing toxicity.^30^^,^^36^^,^^44^ Experimental models also suggest the potential involvement of nonbacterial elements, such as the fungus *Aspergillus rambellii*, in promoting CRC development.^37^

Despite the growing scientific interest in the relationship between gut microbiota and CRC, the existing literature predominantly concentrates on a limited set of well-known microbial taxa. This stands in contrast to the extensive array of gut microbiota components that can influence CRC tumor development, progression, and treatment response. In light of this context, our goal is to delve into the potential of metagenomics as a valuable tool for comprehending CRC and its clinical applications. We aim to explore how metagenomic approaches can offer insights that are not only scientifically enriching but also beneficial in the clinical management of CRC.

## Microbiome profiling techniques

### History of metagenomics

*Metagenomics* involves the genetic examination of microorganisms through the direct retrieval and cloning of DNA from a collection of microorganisms.^38^ The word was initially coined by Handelsman in 1998.^38^

In the late 1970s, Carl Woese introduced the concept of using ribosomal RNA (rRNA) genes as molecular markers for life classification. This, combined with the Sanger automated sequencing method, revolutionized microorganism research, but many questions remain unanswered.^39^ Sanger sequencing was crucial in early microbial community studies but was replaced by next-generation sequencing technologies in 2005. Next-generation sequencing allows parallel sequencing of millions of DNA molecules with variable yields and lengths, making it versatile and impactful.^40^ Next-generation sequencing platforms such as Roche and 454 pyrosequencing, Illumina and Solexa sequencing, and Applied Biosystems and SOLiD detect light signals produced during nucleotide incorporation.^41^ They follow a similar process, which includes DNA extraction, library construction, DNA template preparation, and automated sequence analysis.^42^

The Human Microbiome Project established in 2008^43^ used DNA sequencing technology to study the human microbiome’s impact on health and disease.^45^ The Human Microbiome Project analyzed nearly 3000 bacterial isolates from various body locations, using these as reference genomes for subsequent shotgun metagenomic analysis.^46-48^

### Metagenomics in CRC: 16S rRNA platform

Microbiome research, initially reliant on cultivation-based techniques for understanding gut bacteria and CRC risk,^49^ underwent a transformative shift in the 1990s with the advent of 16S rRNA gene sequencing.^50^ This culture-independent profiling of gut microbial communities played a pivotal role in analyzing fecal samples from CRC patients, unraveling the microbiota’s crucial role in CRC pathogenesis.^51-58^

In 1971, a study failed to establish a link between human gut flora and colon cancer risk.^52^ However, Moore et al. discovered associations between 13 bacterial species and colon cancer risk, particularly in Western diets.^49^ In 2011, Sobhani et al.^52^ found an increased diversity of *Clostridium leptum* and *C. coccoides* subgroups in CRC patients and those with polyps, while Marchesi et al.^58^ detected a statistically significant shift in microbiota composition, with variations in bacterial genera depending on the disease status.

During the 2000s and early 2010s, 16S rRNA surveys exposed a dysbiotic imbalance in the gut microbiota of CRC patients.^51-58^ Pathogenic bacteria, such as *Fusobacterium* and *B. fragilis*, were found to be enriched in CRC patients, while potentially beneficial *Roseburia* bacteria were notably reduced.^52^^,^^53^^,^^56^^,^^59^^,^^60^ These revelations unveiled a dysbiotic imbalance, with an increased presence of oral and opportunistic pathogens and a decrease in symbionts and butyrate producers.^52^^,^^54-59^^,^^61^

Noteworthy discoveries unveiled microbial biomarkers of CRC status, including increased *F. nucleatum*, decreased species of the genus *Roseburia*, and a higher *Bacteroidetes*:*Firmicutes* ratio.^52-54^^,^^56^^,^^59^^,^^60^ Furthermore, they hinted at the role of the microbiota in CRC pathogenesis through mechanisms such as inflammation, gene transfer, and impaired immune regulation.^55-62^ The study used 16S rRNA sequencing to identify microbiome dysbiosis linked to colorectal tumorigenesis, establishing the identification of microbiome dysbiosis linked to colorectal tumorigenesis in a culture-independent manner.^55-62^

In summary, the culture-independent profiling of gut microbiota through 16S rRNA sequencing revealed associations between specific microbes and CRC development, providing new avenues for prevention, diagnosis, prognosis, and treatment, based on a deeper understanding of the complex interplay between gut microbiota and CRC.

### Metagenomics in CRC: shotgun sequencing platform

Shotgun metagenomics is a high-throughput sequencing method that analyzes the genomes of all microorganisms in a sample, including those not grown in a lab, to determine taxonomic makeup, functional potential, and recovery of whole microbial communities.^62^ Shotgun metagenomics provides a comprehensive view of a microbiome’s composition in a single DNA sequencing process, encompassing bacteria, archaea, protists, and fungi. It offers more insights than metagenomic analysis based solely on microbial marker gene amplification, allowing for reconstruction of chromosomal sequences and annotation of microorganism functions. It can offer more insights compared with metagenomic analysis based solely on the amplification of microbial marker genes.^48^

A study comparing 16S rRNA amplicon sequencing and shotgun metagenomic sequencing found that shotgun sequencing outperformed 16S in terms of bacterial species richness, diversity, and gene abundance. It identified 4000 bacterial species and provided broader coverage of bacterial genomes and more than 800 000 predicted microbial genes. Despite being cost-effective, 16S sequencing has limitations in species-level analysis and fails to capture complex microbiome genomic potential. The authors suggest employing shotgun metagenomic sequencing with at least 150 bp reads for comprehensive microbial community profiling.^63^

**Figure 1.**
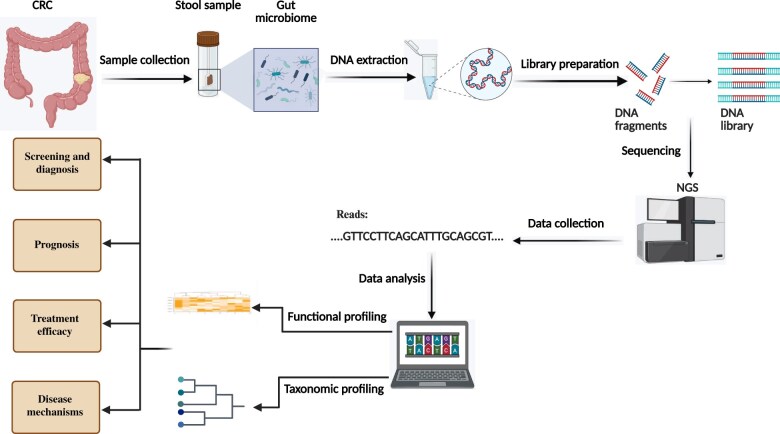
Shotgun metagenomics illuminates gut microbiota-colorectal cancer connections. Shotgun sequencing reveals colorectal cancer–associated taxa, functions, and biomarkers enabling microbiome-based prevention, diagnostics, stratification, and therapy. Abbreviations: CRC = colorectal cancer; NGS = next-generation sequencing.

In summary, 16S rRNA sequencing offers a broad overview of microbial community composition (Figure 1) but lacks species-level analysis and genomic potential compared with whole genome sequencing techniques, requiring specific research goals and resources.

## Clinical perspectives of metagenomics in CRC

### Screening and diagnosis

CRC remains one of the most prevalent and lethal cancers globally. Although current screening modalities like colonoscopy and fecal occult blood tests have proven effective for CRC prevention and early detection, they continue to face substantial barriers to widespread adoption and use. Recent scientific advances suggest analyzing features of an individual’s gut microbiome could offer a novel noninvasive approach to CRC screening with high accuracy.^64^

Several studies have identified microbial biomarkers associated with precancerous lesions like adenomas, suggesting that the microbiome could be used for early CRC screening. However, dysbiosis and specific bacteria have been linked to adenomas and differentiated from healthy controls participants, suggesting that the microbiome could also be used to identify individuals at risk of developing CRC.^65^^,^^66^

A few studies showed promise in detecting precancerous lesions like advanced colorectal adenomas. Combining microbiome and metabolomics data identified biomarkers that outperformed standard screening tests like fecal occult blood tests for diagnosing advanced adenomas.^67-69^

Analyses integrating microbiome, metabolomic, and other data have led to accurate detection of early stage CRC. Moreover, combining microbial and metabolic features improved early CRC diagnosis over standard tests like fecal occult blood tests.^70^ Machine-learning models applied to gut microbiome profiles have shown promise in identifying adenomatous lesions and early stage CRCs. For example, models could differentiate adenoma and early CRC from individuals with area under the curve (AUC) over 80%.^71^^,^^72^ Specific microbes have been associated with the initiation and progression of CRC, representing possible markers of precancerous lesions. *C. spiroforme* and *Tyzzerella nexilis* are 2 bacterial species that are correlated with the initiation and progression of CRC and may be candidates for the detection of CRC, including precancerous lesions.^73^ Although bacteria like *F. nucleatum*, *B. fragilis*, and *Prevotella intermedia* were linked to adenomas and carcinomas, other studies have identified different microbial profiles associated with different stages of CRC.^74^^,^^75^ Studies have found microbiome alterations not just in tumors but also in surrounding healthy tissue, supporting assessment of overall CRC risk. In contrast to stool tests, which may be affected by dietary factors, microbiome analysis of tissue samples could provide a more accurate assessment of CRC risk.^67^^,^^74^ CRC-associated microbiome changes have been detected in stool samples, highlighting potential for noninvasive screening. Furthermore, stool-based tests combining microbial and metabolic markers outperformed standard stool tests like fecal occult blood tests.^73^^,^^76^ Microbial biomarkers identified in one population have proven accurate for detecting early stage CRCs in other cohorts and have been validated across diverse geographical regions.^71^^,^^72^ Analysis of microbiome in extracellular vesicles from urine also showed promise for noninvasive diagnosis.^37^^,^^66^^,^^74^^,^^77-84^

Specific microbial species have been identified that correlate with CRC presence and severity and can serve as predictive biomarkers. *F. nucleatum*, *Parvimonas micra*, *B. fragilis*, and *P. intermedia* are among the top microbial predictors consistently associated with CRC and validated across diverse populations.^7^^,^^37^^,^^66^^,^^72^^,^^74^^,^^75^^,^^77-84^ Other bacteria consistently linked to CRC include *Peptostreptococcus stomatis*,^13^^,^^37^^,^^66^^,^^74^^,^^77-96^  *Porphyromonas spp.*,^85^^,^^86^^,^^92^^,^^95^ and oral pathogens like *Campylobacter rectus*.^86^^,^^93^^,^^96^

Analyses integrating microbial, metabolomic, and host gene expression data have led to highly accurate predictive models for distinguishing CRC cases from healthy control participants. Multi-omics signatures combining microbial and metabolic biomarkers have achieved AUCs over 90% for CRC diagnosis.^87^^,^^88^ Multikingdom models incorporating bacterial, fungal, archaeal, and viral signatures showed promise but require further validation across geographic cohorts.^7^^,^^70^^,^^72^^,^^78^^,^^97^

Table 1 provides an overview of key studies from 2014 to 2023 evaluating fecal microbiome profiles, including bacterial, viral, fungal, and other biomarkers, to distinguish CRC patients from healthy controls across diverse geographic regions. It highlights the sample size, country, specific microbial signatures examined, and diagnostic accuracy in terms of AUC for each study (Table 1). These studies were designed to evaluate the generalizability of microbial signatures across diverse populations, with the objective of developing universally applicable diagnostic tools for CRC.

**Table 1.** Accuracy of fecal microbiome signatures for distinguishing CRC from healthy individuals in global cohorts

Year	Country/Sample size	Signature	AUC	Reference
2014	France/156	Bacterial: 22 species	87%	^13^
Validation cohort: Germany/38 CRC patients -Multi-country/297 control Participants—AUC: 82%				
2018	China, Austria, USA, Germany, France/526	Bacterial: 7 species	80%	^77^
2018	USA/166	Viral: 22 taxa	80%	^65^
Validation cohort participants: Chinese/223, Austrian/109, French-German/157—AUC: Chinese: 76%, Austrian: 73%, French-German: 71%				
2019	China/165	Fungal: 14 biomarkers	CRC vs control: 93% Early CRC vs control: 91%	^98^
Validation cohort participants: China (V1)/420, Germany and France (V2)/198—AUC: CRC vs control: 82% (V1), 74% (V2), early CRC vs control: 81% (V1), 72% (V2)				
2019	Italy/80	Non-coding RNAs: 32 human microRNAs and bacterial small RNAs	87%	^70^
2020	China/330	Archaea: 9 species	82%	^88^
Validation cohort participants: China/420—AUC: 83%				
2020	China/107	Microbial gene: 22 gene	99%	^74^
Validation cohort participants: China/86—AUC: 90%				
2020	South Korea/72	Combination: 2 metabolites and 2 microbial genera	95%-100%	^72^
2021	Austria, Italy, Japan/118	Single-nucleotide variation: 22 markers	75.35%	^96^
Validation cohort participants: China/20—AUC: Validation cohort: 88.02, Resampling validation: 73.08%-79.53%				
2021	China, Austria, Japan/317	Bacteriophage: 5 biomarkers	86%	^99^
2022	Japan, China, USA, Italy, Germany, Austria/172	Bacterial	81%	^78^
2022	Austria, France, Germany, China, Japan, USA, Italy, China/1368	Combination: 11 bacterial, 4 fungal, 1 archaeal species	83%	^79^
2022	Japan/156	Combination: 27 bacterial, 4 fungal and 1 archaeal species	81%	^80^
Validation cohort participants: France, Italy, Austria, Germany, China, Japan, USA, Spain/985—AUC: 75%				
2022	USA, China, France/534	Bacterial: 21 species	62%-78%	^81^
2022	8 cohorts across different geographical regions (USA, China, France, Japan, etc)/2658	Combination: 5 fungal and 9 bacterial species	Fungal: 90.02% Bacterial: 83.22%	^37^
2023	China/441	Combination: 32 microbiomes, 59 KO gene, 16 metabolites	LO-CRC vs control 87% EO-CRC vs control: 91.65%	^14^
Validation cohort participants: China/108—AUC: 78.47%				
2023	China, Japan, Austria, France, USA/1123	Plasmid: 21 biomarkers	Plasmid: 70% Bacterial: 39 species: 79% Combination: 13 plasmids and 37 bacterial species: 84%	^100^

Abbreviations: AUC = area under the curve; CRC = colorectal cancer; EO = early-onset; KO = KEGG orthology; LO = late-onset; Ref = reference; V1 = Validation cohort 1; V2 = Validation cohort 2.

The results demonstrate fecal microbiome signatures can differentiate CRC from healthy control participants with AUC consistently over 80% and up to 99% in some studies. Microbial biomarker panels retained accuracy when validated in independent cohorts, underscoring their potential as noninvasive screening tests. In some studies, the AUC values for validation cohorts are comparable with or even exceed those of training cohorts, which may be attributed to factors such as greater homogeneity within the validation population, the use of robust model development methods, or overfitting in smaller training datasets. Additionally, the range of AUC values reported (eg, Casimiro-Soriguer et al.^75^) likely reflects performance variability across different validation scenarios, including cross-validation folds or geographic cohort comparisons. However, further large-scale validation across populations is still needed before translation into clinical practice. Overall, Table 2 illustrates the promise of stool-based microbiome tests for improving CRC screening globally. Table S1 summarizes microbiome signatures from various published studies that have potential utility as biomarkers for CRC screening and diagnosis. Table S1 provides an overview of the different types of microbiome features that have been associated with CRC status in previous research.

**Table 2.** Signatures for noninvasive CRC progression across global cohorts

Year	Country/Sample size	Distinguish	Signature	AUC	Reference
2015	Austria/156	Carcinoma/Adenoma classification	MLGs: 15 for carcinoma classification	Carcinoma: 98.34%	^112^
			10 for adenoma classification	Adenoma: 87.38%	
Validation cohort: Austria/9—AUC: carcinoma classification: 96% - adenoma classification: 59.56%					
2018	USA/44	Low vs high stage CRC	Microbial: 31 taxa	77.5%	^111^ ^a^
2019	Japan/373	Stage III/IV, SIII/IV Stage 0, S0	Combination: bacterial, metabolite, KO gene	Stage III/IV, Stage III/IV: 85%	^7^
				Stage 0, S0:78%	
2021	China/43 and China, USA, Canada, France/1056	CRC vs AA	Microbial:	26 markers: 89%	^67^
			26 and 11 markers	11 markers: 80%	
2022	China/52	CRC vs AA	15 viruses	Viruses: 81%	^68^
			SNP microbial abundance	SNP: 92%	
				Microbial abundance: 86.5%	
2022	7 datasets/1042	CRC vs AA	Interpretable machine-learning model	81%	^75^
2023	China/750	AA vs control	Single-nucleotide variants: 36 biomarkers	87%	^69^
Validation cohort: China, Austria, France, Italy, Japan, USA—AUC: not reported.					

Abbreviations: AA = Advanced Adenoma; AUC = area under the curve; CRC = colorectal cancer; MLG = metagenomic linkage groups; SNP = Single-nucleotide polymorphism.

a All samples in the studies were fecal except for Huang et al.^117^, which was a tissue sample.

In summary, several studies demonstrate the potential of gut microbiome analysis to detect colorectal tumors at early stages, including precancerous adenomas. Microbiome-based tests may enable earlier diagnosis and prevent progression to late-stage colorectal cancer. However, larger validations are needed before translation into clinical practice.

### CRC treatment efficacy and metagenomics

A growing body of research is elucidating connections between the gut microbiome composition and treatment outcomes in CRC patients. A metagenomic sequencing study of low-set rectal cancer patients found that the composition of the gut microbiota was associated with FOLFOX treatment response. Key gut bacteria associated with response to FOLFOX treatment included *C. ramosum* and *Clostridiales bacterium* 1.7.47 FAA, both of which were present at higher levels in responders both before and after treatment. In contrast, *C. ramosum* was detected in less than 90% of nonresponders after treatment. *Megamonas rupellensis* and *Coprobacter fastidiosus* increased in nonresponders after treatment. The findings suggest baseline gut microbiota patterns may predict FOLFOX response, and the composition changes more drastically during treatment in responders.^101^

Shotgun metagenomic sequencing of fecal samples from 20 CRC patients receiving irinotecan (CPT-11), a common CRC drug, revealed that specific bacterial enzymes, including those from uncultured *Clostridium* spp., *Faecalibacterium prausnitzii*, and a *Bacteroides* spp., were linked to high drug metabolism. Inhibiting these enzymes could potentially reduce adverse drug responses to irinotecan in specific patient groups. Overall, microbiome and metabolomic analyses may enable personalized predictions of irinotecan toxicity and efficacy. Metagenomic analysis of the gut microbiome, in combination with metabolomics, may be used to develop markers for predicting treatment outcomes in CRC.^102^

Another study analyzed the gut microbiome in 7 CRC patients pre- and post-XELOX chemotherapy using 16S rRNA sequencing.^103^ At the phylum level, *Actinobacteria* increased clinically insignificantly after treatment. At lower taxonomic levels, only the order *Bifidobacteriales*, family *Bifidobacteriaceae*, and genus *Bifidobacterium* showed significantly higher abundance after chemotherapy. Specifically, *B. longum* increased markedly. Patients with stable disease had greater increases in *B. longum* vs those with progressive disease, pointing to a potential positive influence.^104^

Finally, in a mouse model, fecal microbiota transplantation (FMT) combined with antiprogrammed cell death 1 immunotherapy enhanced control of colorectal tumors vs either treatment alone. Metagenomic analysis revealed that FMT increased specific bacteria like *B. thetaiotaomicron*, *B. fragilis*, and *B. cellulosilyticus* and decreased *B. ovatus* in the gut microbiome of antiprogrammed cell death 1 treated mice. It also altered microbial gene pathways and metabolites like punicic acid in a potentially antitumor manner. This highlights the promise of modulating the microbiome to improve immunotherapy outcomes.^105^

Table 3 provides an overview of key studies from 2017 to 2022 evaluating associations between the gut microbiome composition and treatment outcomes in CRC patients. It highlights the patient sample size and type, treatment examined, and main findings related to the gut microbiota’s role in influencing chemotherapy response, adverse effects, and immunotherapy efficacy. Key findings demonstrate specific gut bacteria are linked to chemotherapy metabolism and toxicity, composition changes correlate with stable disease vs progression, and fecal microbiota transplantation alters the microbiome in an antitumor manner when combined with immunotherapy in mice. Overall, Table 3 illustrates the growing evidence for the gut microbiome’s ability to affect CRC treatment outcomes, underscoring the microbiome’s potential as a modulator of drug efficacy and toxicity as well as a target for improving therapeutic response.

**Table 3.** Key studies demonstrating the gut microbiome’s impact on colorectal cancer treatment efficacy and toxicity

Patients/Number	Sample type/Treatment	Key findings	Reference
Rectal cancer/37	Fecal/Before and after FOLFOX chemotherapy	-Responders Before and after treatment: Higher levels of *Clostridium ramosum* and *Clostridiales bacterium* 1.7.47 FAA -Nonresponders Post-treatment: *C. ramosum* detected in <90% After treatment: Increases in *Megamonas rupellensis* and *Coprobacter fastidiosus*	^102^
CRC/20	Fecal/Before and after irinotecan chemotherapy	-Bacterial enzymes from uncultured *Clostridium* species, *Faecalibacterium prausnitzii*, and *Bacteroides* species linked to irinotecan metabolism -Inhibiting these enzymes could reduce adverse drug reactions	^103^
CRC/7	Fecal/Before and after XELOX chemotherapy	-Increase in *Bifidobacterium* after treatment -Increase in *B. longum*, especially in patients with stable disease	^104^
Mouse model	FMT with antiprogrammed cell death 1 immunotherapy for CRC	-FMT increased *B. thetaiotaomicron*, *B. fragilis*, *B. cellulosilyticus* -FMT decreased *B. ovatus* -Altered microbial pathways and metabolites	^105^

Abbreviations: CRC = colorectal cancer; FMT = fecal microbiota transplantation.

In summary, emerging research in CRC patients and mouse models demonstrates the gut microbiome can influence chemotherapy efficacy, toxicity, and immunotherapy response. Further large-scale studies validating specific bacterial signatures and evaluating microbiome modulation approaches like FMT will provide more definitive answers. Overall, the microbiome represents a promising new direction for improving CRC treatment.

### CRC progression and metagenomics

Longitudinal studies tracking individuals over time have observed microbiome compositional changes associated with advancing CRC stage and tumor growth. These changes include increasing abundances of *Fusobacteria*, *Porphyromonas*, and *Parvimonas*, which have been linked to worsening stage and tumor progression.^101^ Metagenomic analyses have revealed enrichment of pathogenic virulence factors in more advanced CRC stages, including bacterial adhesins, siderophores, and genotoxins like colibactin.^7^^,^^76^^,^^106-108^ CRC patients exhibited increased *Methanosarcina* and *Shigella* vs adenoma patients.^87^ This implies the microbiome progresses along the adenoma-carcinoma sequence.^107^^,^^108^

A few studies observed microbiome differences between early and late-stage cancers.^91^^,^^95^^,^^109^  *Fusobacterium* and *Leptotrichia* increased in advanced vs early CRC.^109^ Sulfide-producing bacteria were enriched in stages III-IV CRC.^91^ This points to compositional changes with advancing disease. One study noted shifts in the abundance of specific bacteria across progressive stages of CRC. Certain species like *Parvimonas micra* and *F. nucleatum* increased continuously from polyps to late carcinomas.^95^ This underscores microbes that may drive disease advancement. Specific microbes, such as *F. nucleatum*, *P. gingivalis*, and *B. fragilis*, have been repeatedly associated with late-stage colorectal tumors across geographical regions.^95^ Studies categorizing CRCs by molecular subtype have linked certain bacteria to more aggressive consensus molecular subtypes. For example, consensus molecular subtype–1 showed enrichment of *Fusobacteria* and *Bacteroidetes* compared with other subtypes. *F. hwasookii* and *P. gingivalis* were strongly linked to poor prognosis consensus molecular subtype–1.^7^^,^^76^^,^^106-108^ Analyses of CRC microbiomes have revealed disruptions between co-occurring microbes that normally interact in healthy networks, suggesting ecosystem collapse as CRC advances.^110^ Some studies reported changes in the microbiome following cancer treatment. CRC-associated bacteria like *P. stomatis* decreased after tumor removal.^107^ This highlights the microbiome’s dynamic responsiveness to interventions.^7^^,^^69^^,^^76^^,^^78^

Integration of longitudinal microbiome data with corresponding tumor mutational profiles has enabled prediction of CRC stage and identification of stage-specific microbial associations.^95^ A few studies revealed microbiome changes occurring in early stages of carcinogenesis. Differences in bacterial abundances distinguished colorectal adenomas from healthy individuals.^111^ Machine-learning models applied to microbiome functional profiles could differentiate late vs early stage CRCs based on differential microbial activities between stages.^90^ Dysbiosis was observed in multiple polypoid adenomas and intramucosal carcinomas compared with patients.^75^^,^^111^ Combining metabolomics and microbiome data could detect precancerous advanced adenomas.^95^ Comparisons of the microbiome between adenomas, carcinomas, and advanced CRCs have traced taxonomic and functional changes associated with initiation vs progression.^70^ In addition, early onset CRC had increased *Streptococcus gallolyticus* compared with late-onset disease.^68^^,^^107^^,^^112^  Table 2 provides an overview of key studies from 2015 to 2023 evaluating the accuracy of microbiome profiles to distinguish progression from precancerous lesions to late-stage cancer. It summarizes the sample size, country, specific microbial and other signatures examined, and diagnostic accuracy in terms of AUC for each study cohort. It shows that microbial biomarkers from fecal samples consistently distinguish CRC with AUC greater than 80%, even when validated across geographically distinct populations (Table 2). The substantial reduction in AUC for adenoma detection in validation cohorts^112^ may result from the subtler microbiome alterations associated with adenomas compared with CRC and from potential differences in cohort characteristics or sample processing techniques. Table S1 provides a comprehensive summary of microbiome patterns identified in diverse published studies, serving as potential biomarkers for CRC progression (see Table S1). This demonstrates the potential of stool-based microbiome tests as noninvasive screening approaches for detecting CRC advancement. However, further large-scale validation is still needed to translate these microbiome signatures into clinically viable prognostic tests. In summary, a few studies provide clues into microbiome changes along the progression from precancerous lesions to late-stage cancer. Larger longitudinal studies tracking microbiome trajectories within individuals over time are needed to elucidate microbiome contributions to CRC advancement. This knowledge could identify microbial drivers of progression and lead to novel therapies targeting the microbiome.

## Understanding disease mechanisms and metagenomics

Studies have uncovered associations between specific microbes enriched in CRC and production of genotoxic compounds like colibactin, highlighting potential carcinogenic mechanisms.^14^ Analyses of microbial co-occurrence patterns have revealed disrupted mutualism between bacteria and archaea in CRC, suggesting one mechanism by which dysbiosis may contribute to oncogenesis.^91^ Observations of increased horizontal transfer of virulence genes between *Fusobacterium* strains in the CRC microenvironment provide clues into how pathogens may spread cancer-promoting capabilities.^107^^,^^108^ Correlation analysis between CRC-associated microbes and host gene expression has spotlighted potential interactions, such as immune suppression by *Proteobacteria*.^102^ Metagenomic functional profiling has identified microbial genes and pathways enriched in CRC related to functions like iron acquisition, glutathione metabolism, and oxidative stress response.^108^ Integrated meta-omics analysis has traced connections between an altered CRC metabolome, dysbiotic microbiome, and cancer development, highlighting microbiota-metabolite-host interactions.^68^^,^^113^ Species interaction network analysis has revealed collapsed connectivity between cooperative microbes in CRC, probably disrupting microbiome homeostasis.^7^^,^^72^ Machine-learning models have identified CRC-associated microbial genes and activities related to functions like DNA repair, cell cycle control, and proliferation.^7^^,^^69^^,^^76^^,^^78^ Some studies uncovered associations between the microbiome and host pathways and processes involved in CRC. Proteobacteria showed negative correlations with the immune system in adenomas and CRC. Certain bacteria correlated with pathways like MAPK signaling, glutathione metabolism, and glycerophospholipid metabolism.^75^ A few studies linked gut microbes to various CRC-related functions. CRC-associated gene sets were enriched for oxidative stress response, iron transport, and glycine metabolism.^109^ Recent studies underscore how microbial metabolites like oleic acid and allocholic acid can modulate CRC progression through interactions with key tumorigenic pathways such as ENO1 and FXR1, respectively, influencing tumor growth and immune responses.^114^ Furthermore, formate, produced by *F. nucleatum*, has been identified as a protumorigenic metabolite, driving CRC invasion and stemness by triggering AhR signaling.^115^ These findings reinforce the potential of metabolomics in identifying diagnostic biomarkers and therapeutic targets in CRC. Colibactin, produced by some *E. coli*, causes DNA damage promoting carcinogenesis.^93^ One study found evidence of horizontal gene transfer between *Fusobacterium* strains, including virulence factors Fap2 and RadD. The FadA adhesin was identified in diverse *Fusobacterium* spp. This implies complex microbial evolution and sharing of cancer-promoting genes.^89^

Additionally, the microbiome signature is inversely correlated with dendritic cell markers associated with T-cell activation. It is driven by the species *Ruminococcus bromii*, which may induce a partly protective antitumor immune response.^116^

**Figure 2.**
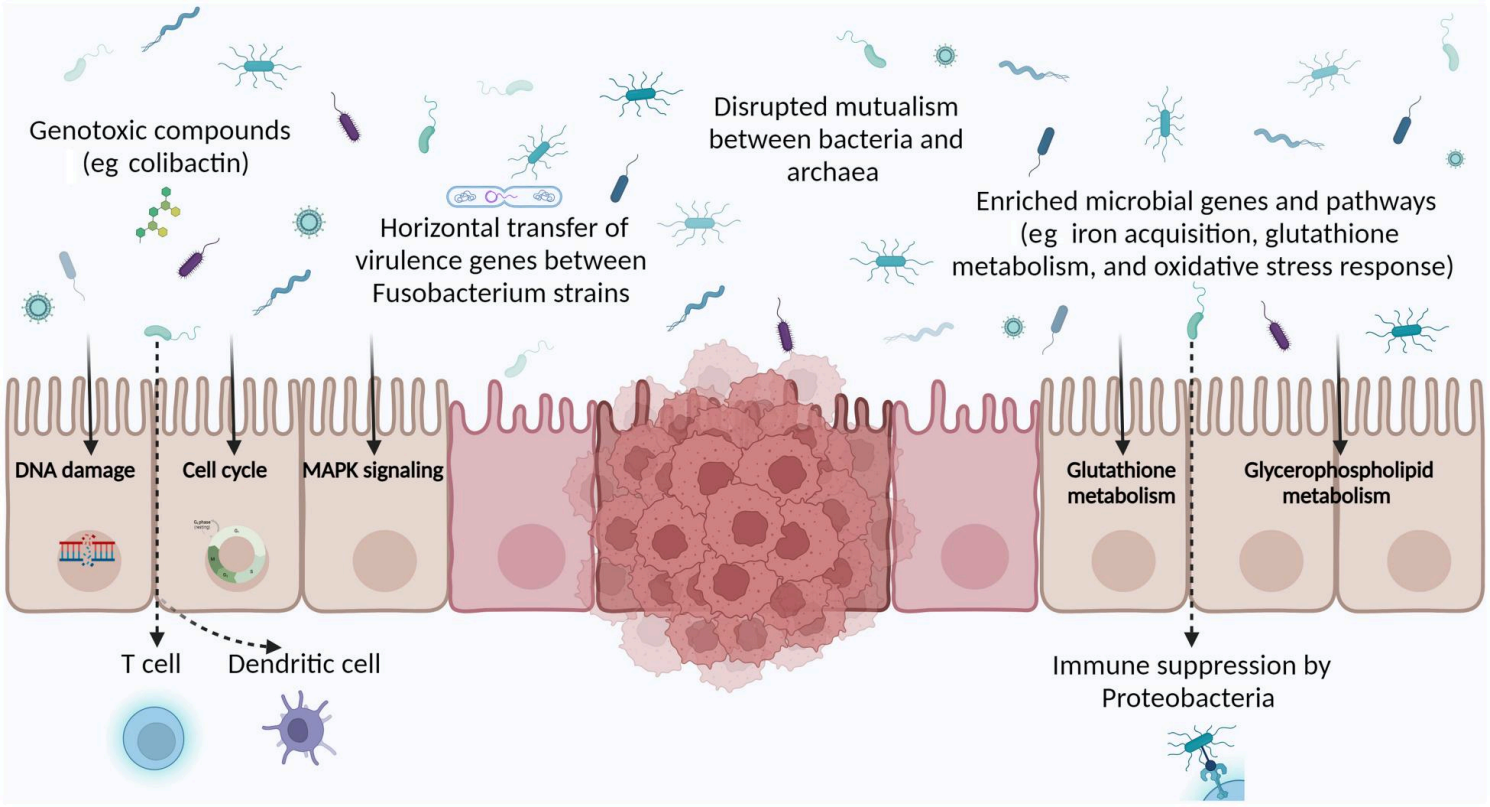
Microbiota-associated mechanisms involved in colorectal cancer pathogenesis. Metagenomics reveals links between CRC-associated microbes and production of genotoxins, horizontal gene transfer, disrupted mutualism, immune suppression, and altered microbial genes/pathways that may promote carcinogenesis as driver or passenger bacteria. These mechanisms contribute to activation of procarcinogenic signaling cascades leading to molecular changes that drive CRC progression. Elucidating these microbe-host interactions has potential for therapeutic or preventive modulation in CRC. Abbreviations: CRC = colorectal cancer.

In summary, a few studies provide preliminary mechanistic clues into how the microbiome may instigate or exacerbate CRC development (Figure 2). Further research is needed to elucidate specific microbial genes and metabolites driving carcinogenesis and understand complex microbe-microbe and microbiome-host interactions underlying CRC progression. These insights can illuminate microbiome-targeted interventions.

## Challenges and future directions

Several challenges remain in elucidating the connections between the gut microbiome and colorectal cancer. A major limitation is that only a small fraction of microbial species have been cultured and sequenced—approximately 80% are considered “microbial dark matter” with unknown functional roles.^117^ Metagenomic sequencing helps bypass the need for culturing but is more expensive than 16S rRNA sequencing, which limits its widespread use.^52^ Additionally, 16S sequencing may miss less abundant but important microbial taxa detected by shotgun metagenomics.^62^ Targeted sequencing approaches may be needed to capture complete profiles. Longitudinal sampling of patients from precancerous adenomas through carcinogenesis is also needed, as early hit-and-run microbes could influence tumorigenesis before being outcompeted by later blooms.^117^ Complementary metabolomic data could provide functional insights into microbial activities.^118^

Although the driver-passenger model provides a useful framework, many open questions remain regarding the specific roles of bacteria across colorectal carcinogenesis. A key challenge is understanding why certain potential keystone pathogens like *F. nucleatum* do not always trigger disease, suggesting complex interplay with host genetics, environment, and the wider microbiota. Further research should clarify if intrinsic factors mediate resistance to dysbiosis in some individuals despite exposure to driver bacteria. Additionally, identifying microbial species that may counteract driver and/or passenger effects and stabilize symbiotic gut communities represents an important goal. Moving forward, integrating findings from gnotobiotic animal models with longitudinal human studies will be critical to elucidate complex bacterial ecosystem dynamics underlying CRC development. Leveraging multi-omics approaches to identify microbial, immunological and genetic factors influencing susceptibility vs resistance to dysbiosis may ultimately enable personalized diagnostics and therapies targeting keystone drivers/passengers.^103^^,^^119^

Utilizing an individual’s gut microbiome for noninvasive CRC screening shows promise, yet several challenges impede clinical implementation. Uncertainty persists regarding the causative role of microbial shifts in tumorigenesis, highlighting the need to establish this link for effective early CRC detection and prevention. Geographical variations in gut microbial composition require attention, necessitating large, multi-ethnic cohort studies with standardized methods to identify generalizable microbiome signatures. Several studies have shown statistically significant geographical and ethnic variations in gut microbiome composition. Black and African American individuals have higher levels of *Fusobacteriota*, including *F. nucleatum*, linked to CRC, while Hispanic and Latino participants exhibit higher *Actinobacteriota* levels.^120^ In regional studies, Japan showed higher prevalence of *Phocaeicola*, *Prevotella*, and *Subdoligranulum*, while China had *Faecalibacterium*, *Mediterraneibacter*, and *Roseburia*.^121^ CRC-associated bacteria like *B. fragilis* and *Subdoligranulum* species were enriched in CRC patients across these regions, and ethnic differences in *Ligilactobacillus* and *Bifidobacterium* were observed in Indian, Chinese, and Malay populations.^122^

Additionally, the lack of unified protocols for microbiome assessment impedes comparisons and external validation, emphasizing the importance of standardized protocols for sample collection, processing, and storage. Optimizing the selection and combination of microbial species, metabolites, and mutational markers into a sensitive screening assay remains a focus of ongoing research before translation into routine noninvasive screening.^64^^,^^123^

Furthermore, there is a notable gap in microbiome studies, with a predominant focus on advanced cancer stages. Investigating microbiome alterations in early lesions like polyps and adenomas could yield better predictive indicators of progression. Technological challenges, including the need for upgraded sequencing coverage and analytical tools, hinder the reliable assembly of complex metagenomes. The prohibitive costs and inconsistent outputs of advanced sequencing platforms limit their adoption in large cohorts. Inconsistencies in microbial patterns across CRC studies result from protocol variability. Standardized protocols for sample collection, processing, and storage are essential to derive generalizable microbial indicators.^124^

The data in this review are drawn from previously published studies, and as such, the authors were not involved in the study design, cohort selection, or data analysis conducted in those works. This lack of direct involvement may limit our ability to fully explain certain variations in AUC values or cohort characteristics. The differences in AUC values reported in Tables 1 and 2 likely stem from variations in study design, cohort composition, and validation methodologies, as outlined in the original studies.

In summary, addressing these challenges through concerted efforts, including multi-omics integrated analyses with robust bioinformatics, is crucial not only for elucidating the complex role of the microbiome but also for clinically validating microbial biomarkers, enabling personalized prevention, and improving the management of colorectal cancer.

## Conclusion

In conclusion, metagenomic approaches have illuminated intricate connections between the gut microbiota and colorectal carcinogenesis. Shotgun sequencing has revealed CRC-associated taxa, functional pathways, and biomarkers with potential clinical utility. Longitudinal sampling has provided clues into microbial trajectories along colorectal tumor progression. Future studies should aim to investigate the reasons behind variability in AUC values, particularly for adenoma detection, and explore methods to improve the generalizability of microbial biomarkers across diverse populations. Although challenges remain, continued multi-omics integration and validation across diverse cohorts will clarify the complex role of microorganisms in instigating and exacerbating CRC. Elucidation of keystone pathogens, mechanistic microbe-microbe and host-microbe interactions, and dysbiosis-mediated effects is imperative. This knowledge can inform microbiome-based prevention strategies, diagnostics, patient stratification, and therapies to improve CRC outcomes. In the future, modulating the gut microbiota may open new possibilities for personalized CRC management. However, realization of precision oncomicrobiome medicine will require unraveling the intricacies of this complex ecosystem and host-microbiota interplay in health and disease.

## Supplementary Material

pkaf026_Supplementary_Data

## Data Availability

No new data were generated or analyzed for this review.
