# Supplementary material for: Gut microbiome in colorectal cancer: metagenomics from bench to bedside
Source: JNCI Cancer Spectr. 2025 Mar 5;9(3):pkaf026. doi: 10.1093/jncics/pkaf026 (PMC12105612; doi:10.1093/jncics/pkaf026)
Supplement: pkaf026_Supplementary_Data [file pkaf026_supplementary_data.zip › Supplementary table 1.docx]

Supplementary table 1. Summary of microbiome markers for colorectal cancer

| **Signature** | **Microbes in Signature** | **Ref** |
| --- | --- | --- |
| Bacterial: 22 species | Streptococcus salivarius, Unclassified Ruminococcus sp., Phascolarctobacterium succinatutens, Butyrivibrio crossotus, Dorea formicigenerans, Methanosphaera stadtmanae, Bifidobacterium angulatum, Eubacterium eligens, Clostridium scindens, Eubacterium ventriosum, Bacteroides caccae, Eubacterium rectale, Lactobacillus ruminis, Fusobacterium gonidiaformans, Lactobacillus salivarius, Bacteroides fragilis, Clostridium hylemonae, Clostridium symbiosum, Porphyromonas asaccharolytica, Peptostreptococcus stomatis, Fusobacterium nucleatum subsp. animalis, Fusobacterium nucleatum subsp. vincentii | [13] |
| Bacterial: 7 species | Bacteroides fragilis, Fusobacterium nucleatum, Porphyromonas asaccharolytica, Parvimonas micra, Prevotella intermedia, Alistipes finegoldii, Thermanaerovibrio acidaminovorans | [80] |
| Viral: 22 taxa | Orthobunyavirus,Tunalikevirus, Phikzlikevirus, Betabaculovirus, Sp6likevirus, Sfi21dtunalikevirus, Punalikevirus, Lambdalikevirus, C2likevirus, Mulikevirus, P22likevirus, HP1likevirus, T4likevirus, CMVlikevirus,T7likevirus, P22likevirus, T5likevirus, T3likevirus, Wphlikevirus, Muikevirus, T1likevirus, O13likevirus | [68] |
| Fungal: 14 biomarkers | Aspergillus flavus, Kwoniella mangrovensis, Pseudogymnoascus sp. VKM F-4518, Debaryomyces fabryi, A. sydowii, Moniliophthora perniciosa, K. heavenensis, A. ochraceoroseus, Talaromyces islandicus, Malassezia globosa, Pseudogymnoascus sp. VKM F-4520, A. rambellii, Pneumocystis murina and Nosemia apis | [121] |
| Non-coding RNAs: 32 human miRNAs and bacterial sRNAs | A signature of 32 features including human miRNAs and bacterial sRNAs: 57.7% human miRNAs (19 miRNAs) and 42.4% bacterial sRNAs (13 bsRNAs) | [73] |
| Archaea: 9 species | Haloplanus CBA1113, Halopelagius longus, Halorubrum tropicale, Haloferax mediterranei, Haloarcula species, Halococcus morrhuae, Halococcus salifodinae, Natrinema species J7-2, Halovenus aranensis | [92] |
| Microbial gene: 22 gene | 20 genes were enriched in colorectal cancer patients, while 2 genes were enriched in healthy controls. 8 genes were from Firmicutes phylum: 5 genes were from Clostridium symbiosum, 1 gene was from Solobacterium moorei, 1 gene was from Coprobacillus genus, 1 gene was unknown function from Firmicutes, 4 genes were from Fusobacteria phylum:, 1 gene was from Fusobacterium nucleatum, 3 genes were from Fusobacterium genus, 10 genes were unknown/unclassified, 12 genes had known functional annotations: Quorum sensing (2 genes), Butanoate metabolism (1 gene), Microbial metabolism in diverse environments (1 gene), Acting on sulfur group of donors (1 gene), Ribosome (1 gene), Genetic information processing (1 gene), Type II secretion system (1 gene), Porphyrin and chlorophyll metabolism (1 gene), Replication and repair (1 gene), Acting on peptide bonds (peptidases) (1 gene), Arginine biosynthesis (1 geneCoprobacillus, Burkholderia, Porphyromonas, Paracoccus, Peptoniphilus, Synechococcus, Cyanothece associated with CRC. | [77] |
| Combination: 2 metabolites and 2 microbial genera | A combination of 2 metabolites (leucine, oxalic acid)  And 2 microbial genera (Collinsella, Solanum melongena) | [75] |
| SNV: 22 markers | 22 SNV markers differentiated CRC cases, including: 11 SNVs in Eubacterium rectale, 9 SNVs in Faecalibacterium prausnitzii, 1 SNV each in Bifidobacterium pseudocatenulatum and Bacteroides vulgatus | [100] |
| Bacteriophage: 5 biomarkers | Five bacteriophages were identified as potential biomarkers for colorectal cancer (CRC) - Peptacetobacter hiranonis phage, Fusobacterium nucleatum animalis 7_1 phage, F. nucleatum polymorphum phage, F. nucleatum animalis 4_8 phage, and Parvimonas micra phage. | [122] |
| Bacterial | Fusobacterium nucleatum, Parvimonas micra, Collinsella tanakaei, and Bacteroides uniformis. | [81] |
| Combination: 11 bacterial, 4 fungal, 1 archaeal species | Bacteria (11)  Gemella morbillorum, Parvimonas micra, Ruminococcus bicirculans, Roseburia intestinalis, Fusobacterium nucleatum, Pseudobutyrivibrio xylanivorans, Streptococcus anginosus, Eubacterium eligens, Peptostreptococcus asaccharolytica, Cellulosilyticum lentocellum, Mogibacterium diversum  Fungi (4)  Aspergillus rambellii, Sistotremastrum suecicum, Talaromyces islandicus, Aspergillus niger  Archaea (1):  Pyrobaculum arsenaticum | [82] |
| Combination: 27 bacterial, 4 fungal and 1 archaeal species | Bacteria (27 species):  Agathobacter sp000434275, Streptococcus thermophilus, Blautia_A sp900066205, Bifidobacterium bifidum MGYG-HGUT-00213, Dialister invisus, Bacteroides fragilis_A, Parvimonas micra, Alistipes onderdonkii, Alistipes enegalensis, Alistipes_A ihumii, Alistipes_A indistinctus, Clostridium_Q symbiosium, Faecalicatena torques, Subdoligranulum sp002287595,, Ruminococcus obeum, Faecalibacterium prausnitzii, Blautia obeum, Eubacterium ventriosum, Lachnospiraceae sp000413455, Roseburia inulinivorans,, Lachnospiraceae sp001315705, Erysipelotrichaceae sp900266575, Romboutsia ilealis, Eubacterium eligens, Hungatella hathewayi, Coprococcus comes. Fungi (4 species):  Aspergillus rambellii, Sistotremastrum suecicum, Talaromyces islandicus, Aspergillus niger, Archaea (1 species): Pyrobaculum arsenaticum | [83] |
| Bacterial: 21 species | CRC-associated: Clostridium symbiosum, Fusobacterium nucleatum, Ruminococcus torques, Gemella morbillorum, Solobacterium moorei, Parvimonas micra, Clostridium citroniae, Peptostreptococcus stomatis, Parvimonas spp., Porphyromonas asaccharolytica, Alistipes onderdonkii, Prevotella intermedia, Clostridium hathewayi, Clostridium sp. MSTE9, Fusobacterium periodonticum, Campylobacter showae Control-associated: Eubacterium eligens, Eubacterium ventriosum, Eubacterium hallii, Roseburia intestinalis, Roseburia inulinivorans, Bifidobacterium catenulatum, Ruminococcus obeum | [84] |
| Combination: 5 fungal and 9 bacterial species | Fungal species:  Aspergillus rambellii, Erysiphe pulchra, Moniliophthora perniciosa, Sphaerulina musiva, Phytophthora capsica  Bacterial species:  Fusobacterium nucleatum, Parvimonas micra, Gemella morbillorum, Escherichia coli, Bifidobacterium longum, Ruminococcus bicirculans, Bifidobacterium adolescentis, Streptococcus thermophilus, Bifidobacterium bifidum, Streptococcus salivarius, Roseburia intestinalis, Pseudobutyrivibrio xylanivorans, Anaerostipes hadrus | [40] |
| Combination: 32 microbiomes, 59 KO gene, 16 Metabolite | LO-CRC vs LO-Control: 32 species microbiome signature,16 metabolite signature, 59 KO gene signature  EO-CRC vs EO-Control: 49 species microbiome signature, 36 metabolite signature, 59 KO gene signature, | [14] |
| Plasmid: 21 biomarkers | NZ_CP036554.1, NZ_AP023416.1 and 19 other plasmids (full list not provided in the article)  39 bacterial species (full list not provided) Included 10 species previously linked to CRC: Parvimonas micra, Peptostreptococcus stomatis, Prevotella intermedia, Porphyromonas a, saccharolytica, Porphyromonas somerae, Porphyromonas uenonis, Gemella morbillorum, Fusobacterium nucleatum, Roseburia hominis, Roseburia intestinalis | [123] |
| MLGs: 15 for Carcinoma classification 10 for Adenoma classification | 15 MLGs for carcinoma classification and 10 MLGs for adenoma classification | [109] |
| Microbial: 31 taxa | Classification of low vs high stage CRC: Porphyromonadaceae, Paludibacter, Bacteroidaceae, Parabacteroides, Odoribacter, Butyricimonas, Oscillibacter, Clostridiaceae, Flavonifractor, Pseudoflavonifractor, Anaerostipes, Coprococcus, Blautia, Ruminococcaceae, Faecalibacterium, Gemmiger, Subdoligranulum, Lachnospiraceae, Marvinbryantia, Fusobacteriaceae, Sutterella, Neisseriaceae, Eikenella, Campylobacteraceae, Campylobacter, Helicobacteraceae, Helicobacter, Pasteurellaceae, Haemophilus, Cyclobacteriaceae, Algoriphagus | [108] |
| Combination: Bacterial, Metabolite, KO gene | For intramucosal carcinoma (S0):  Species: Atopobium parvulum, Solobacterium moorei, Desulfovibrio longreachensis  KEGG Orthology genes(KO) genes: pheC (phenylalanine metabolism)  For detecting colorectal cancer stages are: Metabolites: Leucine, Valine, Phenylalanine, Succinate  For advanced CRC (SIII/IV):  Species: Parvimonas micra, Peptostreptococcus stomatis, Fusobacterium nucleatum, Peptostreptococcus anaerobius  KO genes: None  Metabolites: N-Acetylglucosamine 1-phosphate, Glycylleucine, CholateSo | [7] |
| Microbial:  26 and 11 markers | 11 microbial markers for adenoma vs control: Christensenellaceae R-7 group sp., Eubacterium coprostanoligenes, Ruminiclostridium 9 sp., Christensenellaceae R-7 group sp., Ruminococcaceae UCG-005 sp., Veillonella parvula, Rothia dentocariosa, Aminipila butyrica, Bacteroides (ASVfd), [Eubacterium] ruminantium group, Erysipelatoclostridium  26 microbial markers for distinguishing adenoma from CRC: Streptococcus thermophilus TH1435, Parvimonas micra, Bacteroides dorei, [Clostridium] scindens, Erysipelatoclostridium ramosum, Blautia sp., [Eubacterium] coprostanoligenes group sp., Lachnospira pectinoschiza, [Ruminococcus] gnavus group sp., Porphyromonas sp. HMSC077F02, Streptococcus infantarius, Bacteroides nordii, Lachnospiraceae UCG-010 sp., Porphyromonas sp. 2007b, Ruminococcaceae UCG−002 sp., Hungatella hathewayi WAL-18680, Blautia faecis, [Eubacterium] ventriosum group sp., Tyzzerella 3 sp., Ruminococcus bromii, Roseburia intestinalis, Roseburia hominis A2-183, Merdibacter massiliensis, Streptococcus, Bacteroides (ASV7e), Lachnospiraceae UCG−010, Porphyromonas, Ruminococcaceae UCG−002 | [70] |
| 15 viruses  SNP Microbial abundance | Mus musculus MEPP, Abelson murine leukemia virus, Streptococcus phage TP2J34, Burkholderia virus BcepC6B, Mus musculus mobilized endogenous polytropic provirus, Staphylococcus phage SPbeta-like, Staphylococcus phage YMC-2011, Staphylococcus virus Sfj19, Cellulophaga phage phi47:1, Avian coronavirus, CRESS virus sp., Circoviridae sp., Lake Sarah2associated circular molecule 12, Peduovirus sp., Punavirus sp. | [71] |
| Interpretable machine learning model | Explainable Boosting Machine (EBM) - a type of generalized additive model (GAM) that combines boosting and feature learning for interpretability. Features used: Functional profiles based on KEGG orthologs and eggNOG orthologous groups rather than taxonomic profiles. This provides directly interpretable features. | [78] |
| Single-nucleotide variants (SNVs): 36 biomarkers | 6 single-nucleotide variants (SNVs) panel best for diagnosis (21 synonymous mutations) | [72] |
